# Electroacupuncture pretreatment attenuates myocardial ischemia/reperfusion injury-induced ferroptosis via the p53/SLC7A11/GPX4 pathway

**DOI:** 10.3389/fphys.2026.1928208

**Published:** 2026-10-12

**Authors:** Jing Mao, Qian-Ru Guo, Yi-Pu Luo, Cong-Fei Liu, Zhen-Yang Cao, Yun-Tao Yu, Wen-Wei Wu, Xin-Yu Luan, Dan-Ning Xu, Hua Wang, Pu Chen, Song Wu

**Affiliations:** 1 Department of Acupuncture-Moxibustion and Orthopedics, Hubei University of Chinese Medicine, Wuhan, China; 2 The First Clinical College of Hubei University of Chinese Medicine, Wuhan, China; 3 Hubei Shizhen Laboratory, Wuhan, China

**Keywords:** cardioprotection, electroacupuncture, ferroptotic biochemical phenotype, myocardial ischemia reperfusion injury, p53/SLC7A11/GPX4 signaling pathway

## Abstract

**Introduction:**

A major side effect of reperfusion therapy after myocardial infarction is myocardial ischemia-reperfusion injury (MIRI). Electroacupuncture (EA) preconditioning has a long history of application in the treatment of cardiovascular diseases. This study aims to investigate whether EA preconditioning alleviates MIRI by regulating the p53/SLC7A11/GPX4 pathway to suppress the ferroptotic biochemical phenotype characterized by iron accumulation and lipid peroxidation.

**Methods:**

Healthy male SPF rats were randomly divided into nine groups: Ctrl, Sham, MIRI, EA, RSL-3+EA, COTI-2+EA, Erastin+EA, COTI-2+Erastin+EA, and COTI-2+Pantethine+EA. Before MIRI induction, EA pretreatment was applied daily at CV4, bilateral ST36, and bilateral PC6 for 20 min (2 Hz, 1 mA) for 7 consecutive days. Pharmacological interventions were administered intraperitoneally once daily for 7 consecutive days before EA stimulation. The MIRI model was established by ligating the left anterior descending coronary artery for 30 min followed by 2 h of reperfusion. Myocardial oxidative stress, ferroptosis-related markers, myocardial injury markers, and cardiomyocyte apoptosis were assessed using corresponding commercial kits, DCFH-DA probe combined with flow cytometry, RhoNox-6 fluorescent probe, Western blot, RT-PCR, ELISA, and TUNEL staining combined with flow cytometry.

**Results:**

EA preconditioning inhibited p53 expression, promoted the activation of the SLC7A11/GPX4 pathway, and thereby alleviated myocardial injury. The morphology of cardiomyocytes in EA-pretreated rats was altered, and the apoptosis rate of cardiomyocytes in these rats was also reduced. In addition, administration of Erastin (SLC7A11 inhibitor) showed effects similar to those of COTI-2 (p53 agonist) intervention, whereas pantethine (SLC7A11 agonist) reversed the effects of COTI-2, exerting anti-ferroptotic and cardioprotective effects.

**Discussion:**

As a proof‑of‑concept study with limited sample size per experimental group, our findings preliminarily suggest that EA preconditioning ameliorates MIRI by suppressing the ferroptotic biochemical signature characterized by myocardial iron overload and lipid peroxidation through the p53/SLC7A11/GPX4 pathway. Further studies with larger sample sizes are still needed for validation.

## 1 Introduction

Under cardiovascular disease-induced myocardial ischemia/hypoxia conditions, such as acute myocardial infarction, heart transplantation, acute heart attack and trauma, the rapid restoration of blood flow and oxygen supply remains the most effective strategy to reduce ischemic injury and limit infarct size (Ibáñez et al., 2015). However, the reperfusion process itself has been demonstrated to cause additional damage to cardiomyocytes, a phenomenon defined as myocardial ischemia/reperfusion injury (MIRI) (Algoet et al., 2023). In current clinical practice, vasodilators (e.g., nifedipine), β-blockers, ACE inhibitors and antiplatelets serve as first-line pharmacological treatments for MIRI. These agents stabilize hemodynamics and reduce infarct size to relieve acute ischemic injury (Ibáñez et al., 2015; Santa-Helena et al., 2019);, which aligns with the TCM strategy of relieving acute manifestations for urgent cases. Nevertheless, such single-target drugs fail to regulate core pathogenic drivers like ferroptosis and persistent oxidative stress, and carry side effects including hypotension, tachycardia and hemorrhage (Hashimoto et al., 2018; Santa-Helena et al., 2019). Although these adverse reactions are clinically manageable, there is an unmet demand for adjunct therapies targeting the root pathological lesions, consistent with the TCM tenet of addressing fundamental root causes for chronic ailments. As a multi-target non-pharmacological preconditioning strategy, EA preconditioning synergizes with conventional drugs to simultaneously achieve acute symptomatic relief and root-cause intervention. Given the absence of mature specific therapies against MIRI, developing novel complementary interventions such as EA preconditioning is clinically imperative.

The pathophysiological mechanisms underlying MIRI are complex and involve Ca2+ overload, mitochondrial energy metabolic dysfunction, oxidative stress, inflammation, immune activation, and metabolic derangements (Wu et al., 2018). As cardiomyocytes are terminally differentiated cells incapable of self-renewal through proliferation, the damaged myocardial tissue is usually replaced by fibrous tissue during the recovery phase following MIRI, resulting in irreversible functional impairment (Hashimoto et al., 2018). Consequently, minimizing cardiomyocyte mortality represents a critical therapeutic objective in managing MIRI. Existing research has established the involvement of multiple cell death pathways in MIRI pathogenesis, including apoptotic processes, regulated necrosis, inflammatory-mediated cell death, and the iron-catalyzed phenomenon of ferroptosis (Yang and Stockwell, 2008; He et al., 2009; Konstantinidis et al., 2012). Notably, ferroptosis—a distinctive form of iron-dependent programmed cell death marked by excessive phospholipid peroxidation—has emerged as a particularly significant focus of investigation (Yang and Stockwell, 2016; Stockwell et al., 2017; Jiang et al., 2021). The elevation of intracellular labile iron pools occurs through mechanisms such as ferritinophagy (selective autophagy of ferritin) and transferrin receptor-mediated iron uptake. These free ferrous ions subsequently engage in Fenton chemistry with hydrogen peroxide, generating highly reactive hydroxyl radicals that initiate lipid peroxidation cascades, ultimately culminating in ferroptotic cell death (Gao et al., 2015; Gao et al., 2016; Hou et al., 2016; Geng et al., 2018; Yambire et al., 2019; Chen et al., 2020; Bao et al., 2021). Concurrently, in the presence of iron, lipids within the cell undergo peroxidation to form lipid hydroperoxides (LOOHs). In the absence of timely catalytic intervention by glutathione peroxidase 4 (GPX4), the peroxidation process may extend to neighboring phospholipids containing polyunsaturated fatty acids (PUFA-PLs) through iron-mediated mechanisms, ultimately causing membrane disruption and cellular demise. As a crucial antioxidant, glutathione (GSH) serves a vital function in shielding cells from oxidative stress by facilitating the removal of reactive oxygen species under the catalytic action of GPX4 (Hu et al., 2020). Mammalian cell membranes harbor the Xc^-^transport complex, comprising two subunits: the light chain (Solute Carrier Family 7 Member 11, SLC7A11) and the heavy chain (SLC3A2). This system facilitates the reciprocal transport of extracellular cystine and intracellular glutamate (Fang et al., 2019). Following cellular uptake, cystine undergoes reduction to form cysteine, which serves as the fundamental building block for GSH biosynthesis. Therefore, the Erastin that specifically inhibits the Xc^-^ system, as well as GPX4 inhibitors and RSL3, could induce ferroptosis (Dixon et al., 2012; Dixon et al., 2014; Yang et al., 2014). Research indicates that the transcriptional activity of SLC7A11 significantly influences the amino acid transport capacity of the Xc^-^ system. Notably, both the tumor suppressor protein p53 and the histone deubiquitinating enzyme BAP1 have been demonstrated to negatively regulate SLC7A11 expression, consequently impairing glutathione biosynthesis and inducing ferroptotic cell death (Jiang et al., 2015; Zhang et al., 2018). In investigations concerning MIRI mediated by ferroptosis, hydroxysafflor yellow A (HSYA), the principal bioactive constituent of safflower in traditional Chinese medicine, was identified as a ferroptosis inhibitor through its activation of the HIF-1α/SLC7A11/GPX4 signaling axis, thereby alleviating acute MIRI in murine models (Ge et al., 2023). Additionally, the citrus flavonoid nobiletin was found to mitigate MIRI in diabetic rats by suppressing the expression of key ferroptosis-related proteins, specifically ACSL4 and NCOA4 (Huang et al., 2023).

In clinical practice, EA preconditioning serves as a safe non-pharmacological cardioprotective intervention with verified efficacy across multiple perioperative scenarios. A multicenter 2-year randomized controlled trial confirmed that EA pretreatment at PC6 and PC4 1–2 h before PCI lowered the incidence of type 4a myocardial infarction and long-term MACCE in coronary artery disease patients (Wang et al., 2015). Another clinical trial proved sequential preoperative EA alleviated post-operative cTnI release and shortened ICU stay for patients receiving heart valve replacement surgery (Yang et al., 2010). For patients with reduced ejection fraction heart failure, long-term EA therapy combined with routine medication persistently improved cardiac function and exercise capacity (Supriyana et al., 2025) Human autonomic nerve studies further illustrated low-frequency EA at pericardium acupoints suppresses excessive cardiac sympathetic activity, providing a physiological basis for its myocardial protective effects (Nakahara et al., 2016).These clinical studies support the translational potential of EA preconditioning as a complementary cardioprotective intervention alongside conventional reperfusion strategies.

Acupuncture preconditioning is derived from the TCM concept of “preventive treatment of disease”, in which appropriate stimulation at specific acupoints is applied before disease onset or aggravation to elicit adaptive stress responses and maintain internal homeostasis. Electroacupuncture (EA), which integrates traditional acupuncture with electrical stimulation, is widely used in the rehabilitation of conditions such as stroke. Moreover, EA has demonstrated therapeutic efficacy in Parkinson’s disease, colitis, allergic airway inflammation, and stress urinary incontinence, among other disorders (Zhu et al., 2020). Growing evidence indicates that EA can effectively alleviate MIRI. Yang et al. reported that EA preconditioning targeting corticotropin-releasing hormone (CRH) neurons in the hypothalamic paraventricular nucleus significantly mitigated myocardial ischemia-reperfusion injury (Zhou et al., 2024). Additionally, EA has been shown to promote the nuclear translocation of Nrf2, thereby activating the Nrf2/SLC7A11/GPX4 pathway, suppressing ferroptosis in neurons after ischemic stroke, and exerting neuroprotective effects (Yang et al., 2025). However, the mechanism by which EA pretreatment attenuates cardiomyocyte ferroptosis and alleviates MIRI remains incompletely understood. This study was designed to investigate whether EA mitigates MIRI by inhibiting ferroptosis in cardiomyocytes through modulation of the p53/SLC7A11/GPX4 signaling pathway.

## 2 Results

### 2.1 Electroacupuncture pretreatment alleviates MIRI by suppressing ferroptosis

We first characterized the independent cardioprotective effect and ferroptosis-suppressing biochemical phenotype conferred by EA pretreatment against MIRI myocardium. To preliminarily verify whether ferroptosis contributes to EA-mediated myocardial protection, we co-administered RSL-3, a ferroptosis inducer, alongside EA pretreatment to examine whether RSL-3 abrogates the cardioprotective benefits of EA.

#### 2.1.1 Electroacupuncture treatment improves energy metabolism and mitigates oxidative damage in cardiomyocytes during MIRI

Myocardial cells rely on mitochondrial oxidative phosphorylation to synthesize large amounts of ATP each day to sustain basal energy metabolism and cardiac pumping function. In the established MIRI rat model, ATP levels in cardiomyocytes were first quantified. Compared with the Ctrl and Sham groups, ATP production was significantly reduced in the MIRI group (**Figure 1A**). Notably, EA intervention markedly restored ATP levels (**Figure 1A**).To further evaluate oxidative stress status, the lipid peroxidation marker MDA and intracellular ROS were measured. Both MDA and ROS levels were significantly elevated in the MIRI group, indicating pronounced oxidative stress in cardiomyocytes (**Figures 1B, C**). Following EA treatment, these oxidative markers were significantly reduced (**Figures 1B, C**). Concurrently, SOD activity was substantially increased after EA administration (**Figure 1D**). Collectively, these findings demonstrate that EA effectively improves impaired energy metabolism and attenuates oxidative damage in cardiomyocytes subjected to MIRI.

**Figure 1:**
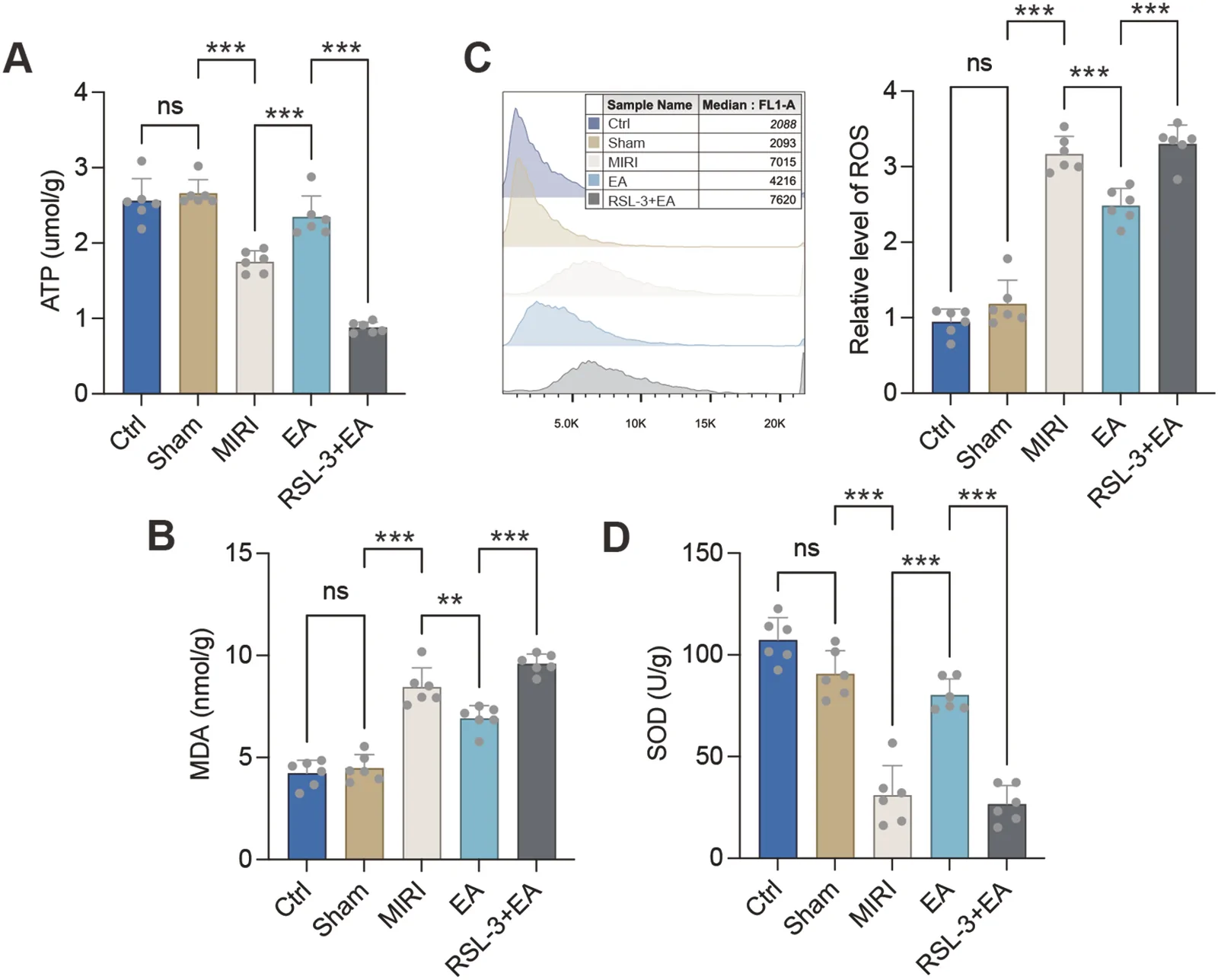
Electroacupuncture treatment improves energy metabolism and mitigates oxidative damage in cardiomyocytes during MIRI. **(A)** The level of ATP in cardiomyocytes was determined through kits; **(B)** Intracellular MDA in cardiomyocytes was determined based on kits; **(C)** The ROS in cardiomyocyte was determined by flow cytometry; **(D)** The SOD in cardiomyocytes was determined based on kits. [Data are presented as Means ± SD of n = 6 independent biological replicates. Intergroup differences were assessed via one-way ANOVA followed by Tukey’s *post-hoc* multiple comparison test; **p < 0.01, ***p < 0.001; ns, not significant (*p*> 0.05)].

#### 2.1.2 Electroacupuncture mitigates the ferroptotic biochemical phenotype and alleviates myocardial iron overload in MIRI

To confirm the involvement of ferroptosis in MIRI, intracellular Fe2^+^levels in myocardial cells were measured. The MIRI group exhibited a marked elevation in Fe²^+^ levels compared with the Ctrl and Sham groups, whereas EA treatment significantly reduced Fe²^+^accumulation (**Figure 2A**). Furthermore, the concentrations of reduced GSH and its oxidized form (GSSG) were quantified. Analysis revealed that cardiomyocytes subjected to MIRI exhibited elevated GSSG levels accompanied by diminished GSH content (**Figures 2B–D**), demonstrating iron-mediated oxidative damage. Subsequent molecular examinations of myocardial specimens were conducted through quantitative polymerase chain reaction (qPCR) and immunoblotting techniques to investigate crucial proteins associated with ferroptosis, specifically transferrin receptor 1 (TFR1), acyl-CoA synthetase long-chain family member 4 (ACSL4), and ferritin heavy chain 1 (FTH1). The MIRI model displayed substantial upregulation of both TFR1 and ACSL4 expression, whereas FTH1 levels were notably decreased, aligning with characteristic ferroptotic features such as augmented iron absorption, elevated lipid peroxidation, and compromised iron storage capacity. Following EA intervention, TFR1 expression was downregulated, thereby reducing transferrin-mediated iron uptake; ACSL4 expression decreased, limiting lipid peroxidation; and FTH1 expression increased, enhancing intracellular iron sequestration. Together, these changes significantly suppressed ferroptotic activity in MIRI rats (**Figures 2E, F**). Overall, these findings demonstrate that EA effectively mitigates ferroptosis in MIRI-induced cardiomyocytes and reduces pathological iron deposition within myocardial tissue.

**Figure 2:**
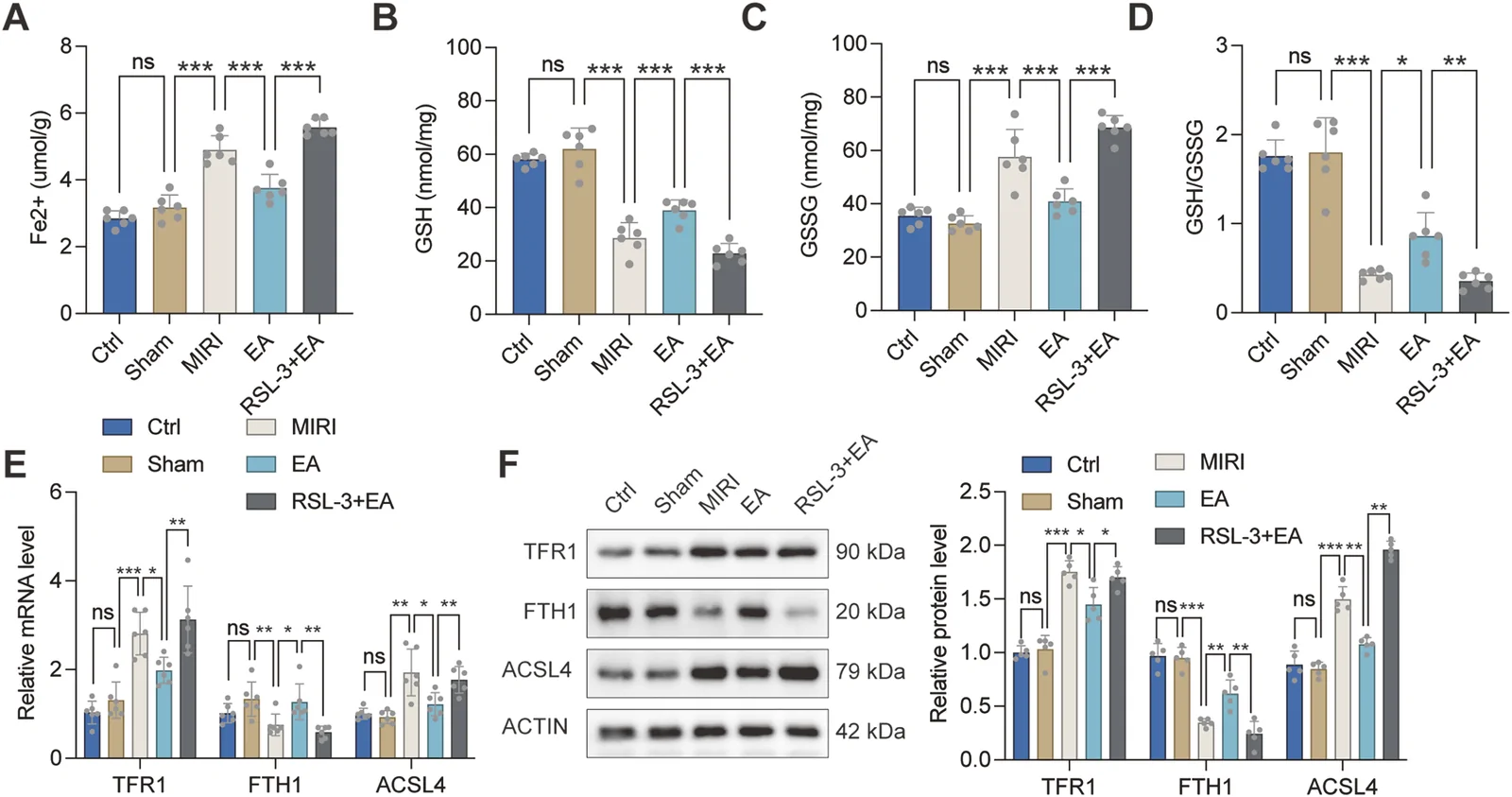
Electroacupuncture mitigates the ferroptotic biochemical phenotype and alleviates myocardial iron overload in MIRI. **(A)** Fe²^+^ levels in cardiomyocytes were measured using a specific kit; **(B–D)** The contents of GSH and GSSG in cardiomyocytes were detected using specific assay kit; **(E)** The mRNA contents of ferroptosis-related proteins (TFR1, FTH1, and ACSL4) were determined via RT-PCR; **(F)** The proteins contents of ferroptosis-related proteins (TFR1, FTH1, and ACSL4) were determined through Western blot. [Data are presented as Means ± SD of n = 6 independent biological replicates. Intergroup differences were assessed via one-way ANOVA followed by Tukey’s *post-hoc* multiple comparison test; *p < 0.05, **p < 0.01, ***p < 0.001; ns, not significant (*p*> 0.05)].

#### 2.1.3 Electroacupuncture alleviates MIRI and preserves cardiac function by restraining the ferroptotic biochemical signature

To evaluate the protective effects of EA on MIRI, plasma samples from each experimental group were analyzed using ELISA to quantify key biomarkers of myocardial injury. Compared to the Ctrl and Sham groups, the MIRI group exhibited significantly increased concentrations of creatine kinase–myocardial band isoenzyme (CK-MB), lactate dehydrogenase (LDH), and cardiac troponin I (cTnI) (**Figures 3A–C**). EA intervention effectively lowered these biomarker levels, suggesting a protective effect against myocardial damage. Evaluation of cardiomyocyte apoptosis was performed using specialized detection kits, with DNA fragmentation analysis conducted through flow cytometry techniques. A notable rise in apoptotic cell percentage was observed in the MIRI group, which was substantially mitigated by EA administration (**Figures 3D, E**). Additional investigations involving Western blot and qPCR revealed that MIRI triggered significant elevation of the apoptosis-promoting protein Bax and suppression of the anti-apoptotic protein Bcl-2. After EA therapy, marked reductions in Bax and Caspase-3 expression were detected, accompanied by normalization of Bcl-2 levels (**Figures 3F, G**), providing further evidence for EA’s capacity to inhibit apoptotic processes in cardiac cells. To assess myocardial tissue injury and infarct size, fresh heart tissues were subjected to TTC and Evans Blue staining. In the staining outcome, white corresponded to infarcted tissue. EA-treated rats exhibited a markedly smaller infarct area and improved tissue viability compared with the untreated MIRI group (**Figure 3H**). Hematoxylin–eosin (HE) staining further supported these findings: the structural disorganization, increased collagen deposition, and myofibrillar disruption characteristic of MIRI were substantially improved after EA treatment (**Figure 3I**). Taken together, these findings demonstrate that EA markedly attenuates MIRI by suppressing ferroptosis and limiting cardiomyocyte damage, ultimately contributing to improved cardiac function.

**Figure 3:**
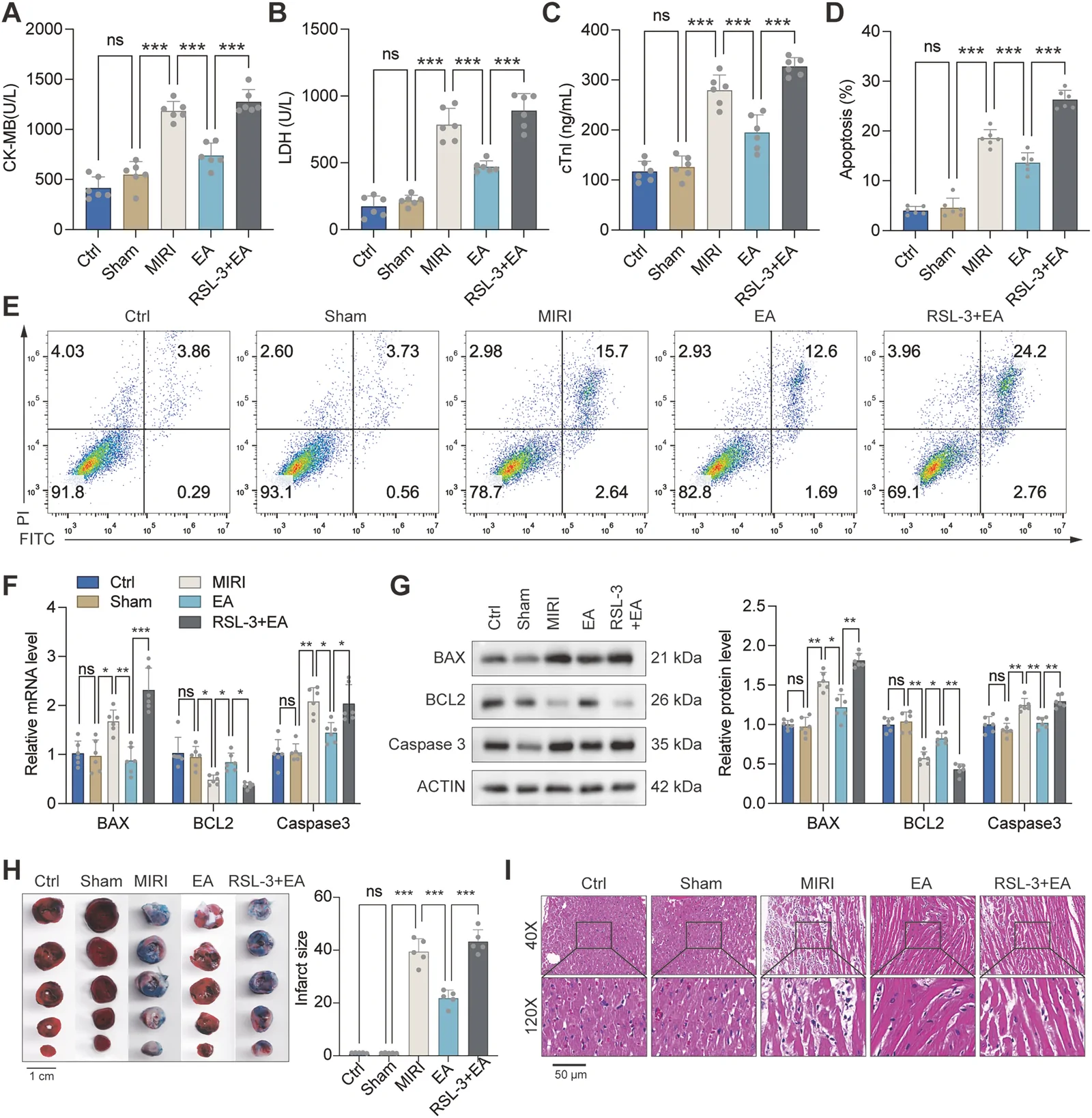
Electroacupuncture alleviates MIRI and preserves cardiac function by restraining the ferroptotic biochemical signature. **(A–C)** Serum from each group of MIRI rat was collected to detect enzymes related to myocardial injury (CK-MB, LDH and cTnI); **(D, E)** Apoptosis of MIRI cardiomyocytes was determined by flow cytometry using TUNNEL staining; **(F)** mRNA levels of proteins associated with apoptosis were identified by RT-PCR (Bax, Bcl2 and caspase3); **(G)** Apoptosis-related protein levels were determined by Western blot; **(H)** Evans Blue-TTC dual staining shows the myocardial infarct size in SD rats; **(I)** Histopathological HE staining of myocardial tissue in each group. [Data are presented as Means ± SD of n = 6 independent biological replicates. Intergroup differences were assessed via one-way ANOVA followed by Tukey’s *post-hoc* multiple comparison test; *p < 0.05, **p < 0.01, ***p < 0.001; ns, not significant (*p*> 0.05)].

### 2.2 Electroacupuncture protects against MIRI via the p53/SLC7A11/GPX4 pathway

Having confirmed the fundamental capacity of EA pretreatment to suppress ferroptotic biochemical phenotypes, we further performed pharmacological rescue experiments targeting the p53/SLC7A11/GPX4 axis. We treated EA-preconditioned MIRI rats with either COTI-2 (a p53 agonist) or Erastin (an SLC7A11 inhibitor) separately to verify whether p53 activation or SLC7A11 inhibition could reverse the protective effects of EA pretreatment against oxidative injury and ferroptosis.

#### 2.2.1 Electroacupuncture enhances myocardial energy metabolism and alleviates oxidative damage via the p53/SLC7A11/GPX4 pathway

Based on the results obtained in this study, the ferroptosis was induced by MIRI in cardiomyocytes of rats. To investigate which specific signaling pathway was involved in the ferroptosis process of MIRI-induced cardiomyocytes, previous studies have demonstrated that p53 could bind to other proteins to inhibit SLC7A11, thereby blocking GSH synthesis and subsequently inducing ferroptosis. In this study, an agonist of the p53 (COTI-2+EA group), COTI-2, and an inhibitor of SLC7A11 (Erastin+EA group), Erastin, were respectively administered to MIRI-EA-pretreated rats to determine whether EA suppressed oxidative damage and ferroptosis in MIRI rats through the p53/SLC7A11 pathway. The results showed that ATP and the antioxidant SOD were significantly downregulated in COTI-2+EA group and Erastin+EA group, while the oxidative markers MDA and ROS were significantly upregulated compared with those in EA-treated MIRI the cardiomyocytes from rats (**Figures 4A–D**). These results demonstrated that EA intervention could improve myocardial energy metabolism and alleviate oxidative damage through the p53/SLC7A11/GPX4 pathway.

**Figure 4:**
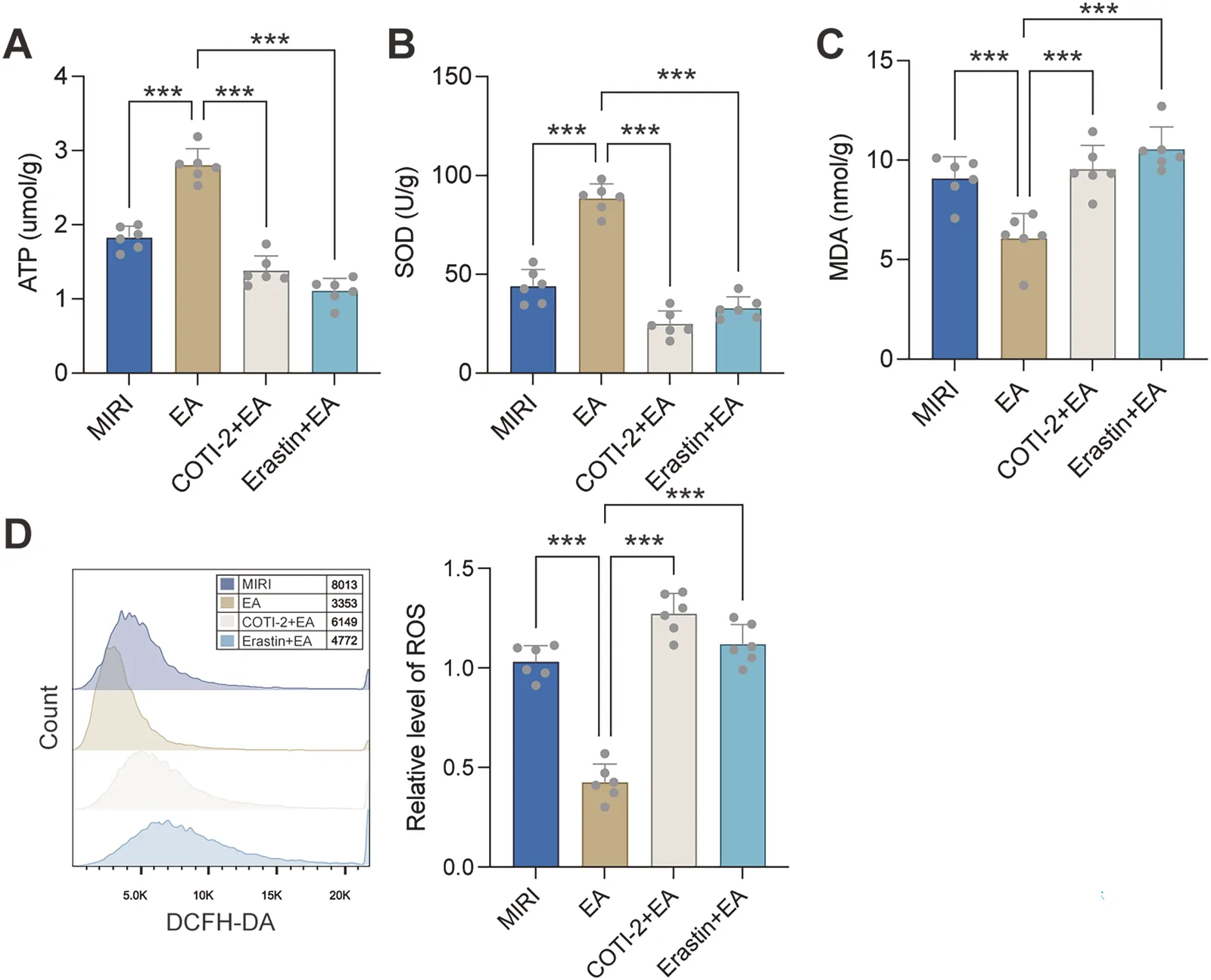
Electroacupuncture enhances myocardial energy metabolism and alleviates oxidative damage via the p53/SLC7A11/GPX4 pathway. **(A)** The content of ATP in cardiomyocytes was determined; **(B–D)** Substances associated with oxidative damage to cardiomyocytes was measured by using kits or flow cytometry (SOD, MDA and ROS). [Data are presented as Means ± SD of n = 6 independent biological replicates. Intergroup differences were assessed via one-way ANOVA followed by Tukey’s *post-hoc* multiple comparison test; ***p < 0.001.]

#### 2.2.2 Electroacupuncture modulates the ferroptotic biochemical phenotype through the p53/SLC7A11/GPX4 pathway

Following EA treatment, cardiomyocytes subjected to MIRI were additionally exposed to either a p53 activator (designated as COTI-2+EA group) or an SLC7A11 blocker (termed Erastin+EA group). Consistent with expectations, relative to the EA-pretreated cells, administration of either the p53 activator or SLC7A11 inhibitor resulted in substantially higher intracellular Fe²^+^ concentrations (**Figure 5A**), along with pronounced increases in GSSG quantities and significant declines in GSH levels (**Figures 5B–D**). Cardiac tissue specimens obtained from all experimental cohorts were analyzed via Western blot and qPCR to quantify the protein expression profiles related to ferroptosis, encompassing p53, SLC7A11, GPX4, TFR1, FTH1, and ACSL4. The results revealed that the COTI-2+EA group displayed markedly upregulated p53, TFR1, and ACSL4 protein levels, while exhibiting downregulated expression of SLC7A11, FTH1, and GPX4. Analogously, in the Erastin+EA group, TFR1 and ACSL4 expression was significantly enhanced, whereas SLC7A11, FTH1, and GPX4 levels were substantially diminished (**Figures 5E, F**). These observations collectively indicate that EA treatment exerts regulatory effects on cardiomyocyte ferroptosis by influencing the p53/SLC7A11/GPX4 signaling cascade.

**Figure 5:**
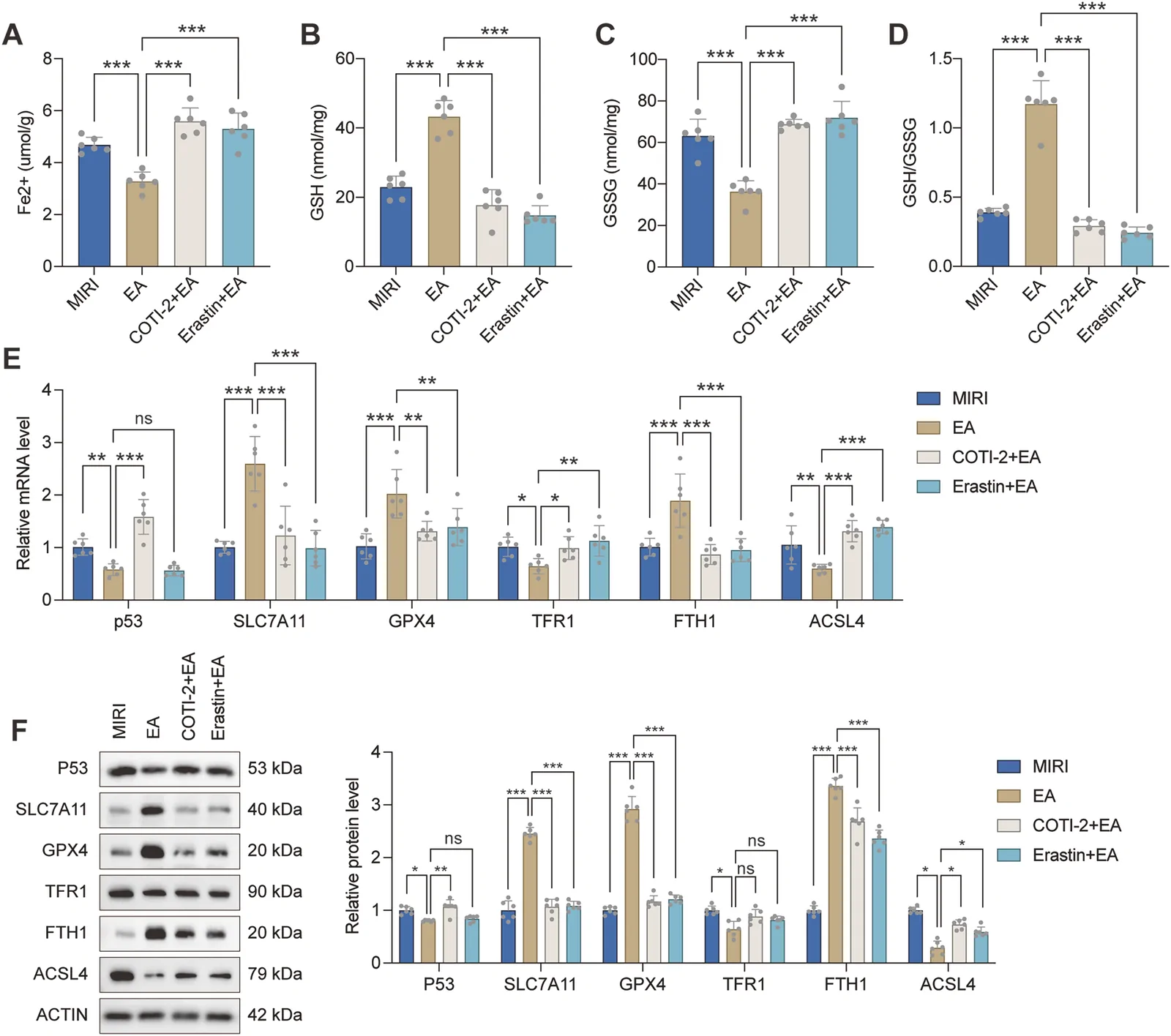
Electroacupuncture modulates the ferroptotic biochemical phenotype through the p53/SLC7A11/GPX4 pathway. **(A)** Fe^2+^ content of cardiomyocytes was determined (EA: MIRI rat were pretreated with EA; P53^+^ group: cardiomyocytes of rat with MIRI were treated with p53 agonist; SLC^-^ group: cardiomyocytes of rat with MIRI were treated with EA and then added SLC7A11 inhibitor); **(B–D)** The kit detected the concentrations of GSH and GSSG in cardiomyocytes; **(E)** RT-PCR detected the mRNA level of ferroptosis-related proteins (p53, SLC7A11, GPX4, TFR1, FTH1 and ACSL4). **(F)** Western blot detected protein levels of ferroptosis-related proteins (p53, SLC7A11, GPX4, TFR1, FTH1 and ACSL4).(Data are presented as Means ± SD of n = 6 independent biological replicates. Intergroup differences were assessed via one-way ANOVA followed by Tukey’s *post-hoc* multiple comparison test; *p < 0.05, **p < 0.01; ***p < 0.001, ns, not significant (*p*> 0.05)).

#### 2.2.3 Electroacupuncture protects cardiomyocytes via the p53/SLC7A11/GPX4 pathway

Plasma samples were collected from rats via ELISA to analyze several enzymes associated with myocardial injury, including CK-MB isoenzyme, LDH and cTnI. Analysis of the experimental data demonstrated that all three enzymes exhibited marked upregulation in both the COTI-2+EA group and the Erastin+EA group when compared to the EA group (**Figures 6A–C**). Flow cytometric assessment of cardiomyocyte apoptosis revealed a substantial elevation in apoptotic cell counts within these experimental groups (**Figure 6D**). Subsequent protein analysis through Western blot and qPCR techniques identified significant alterations in apoptosis-related markers. The COTI-2+EA and Erastin+EA groups displayed increased levels of BAX and Cleaved Caspase-3 proteins, concurrent with a notable reduction in BCL2 expression relative to the EA group (**Figures 6E–H**). According to the Evans Blue-TTC staining (**Figure 6I**) and HE staining (**Figure 6J**), the significantly improved phenotype of myocardial damage in MIRI rats after EA treatment was reversed upon both the COTI-2+EA group and the Erastin+EA group, suggesting that EA protected cardiomyocytes via the p53/SLC7A11/GPX4 pathway.

**Figure 6:**
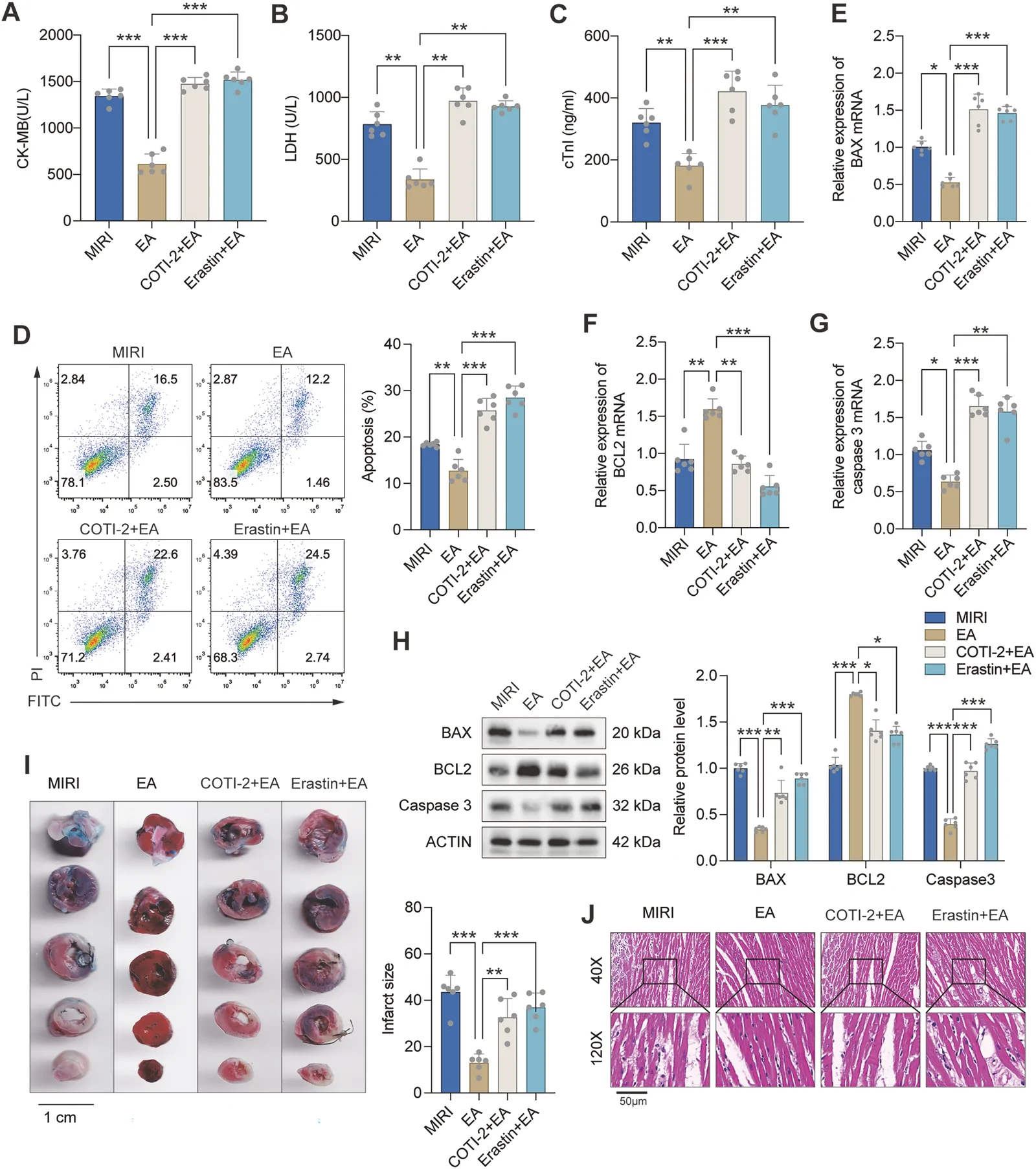
Electroacupuncture protects cardiomyocytes via the p53/SLC7A11/GPX4 pathway. **(A–C)** Myocardial cell injury markers in each group of rat were detected using ELISA kits; **(D)** The apoptosis of rat in each group was detected by flow cytometry; **(E–G)** The mRNA levels of apoptosis-related proteins in cardiomyocytes in each group were identified by RT-PCR;**(H)** The expression of apoptosis-related proteins in cardiomyocytes in each group was quantified by Western blot; **(I)** Evans Blue-TTC dual staining shows the myocardial infarct size in SD rats; **(J)** Histopathological HE staining of myocardial tissue in each group. [Data are presented as Means ± SD of n = 6 independent biological replicates. Intergroup differences were assessed via one-way ANOVA followed by Tukey’s *post-hoc* multiple comparison test; *p < 0.05, **p < 0.01, ***p < 0.001.

### 2.3 The SLC7A11 agonist reverses the pro-ferroptotic effects of the p53 agonist, confirming the p53/SLC7A11/GPX4 pathway as the target of electroacupuncture

To further validate the linear regulatory relationship of the p53/SLC7A11/GPX4 pathway in EA-mediated cardioprotection, we designed combined drug intervention groups. We simultaneously administered COTI-2 (p53 agonist) and pantethine (SLC7A11 agonist) to EA-treated rats, to test whether upregulating SLC7A11 could counteract the pro-ferroptotic injury induced by p53 overactivation, and ultimately confirm the complete pathway logic of EA action.

#### 2.3.1 The SLC7A11 agonist reverses the pro-ferroptotic effects of the p53 agonist, confirming that electroacupuncture protects against MIRI via the p53/SLC7A11/GPX4 signaling pathway

To further validate the involvement of the p53/SLC7A11/GPX4 pathway in the cardioprotective effects of EA, MIRI model rat receiving EA intervention were additionally treated with either a combination of a p53 agonist and an SLC7A11 agonist (COTI-2+Pantethine+EA group), or a p53 agonist and an SLC7A11 inhibitor (COTI-2+Erastin+EA group). The intracellular Fe2+ levels in cardiomyocytes were first measured using commercial assay kits. In the COTI-2+Pantethine+EA group, the increase in Fe2+ content was reversed (**Figure 7A**). Concurrently, increased levels of reduced GSH and decreased levels of oxidized GSSG were observed following administration of the SLC7A11 agonist compared with COTI-2+EA group and COTI-2+Erastin+EA group (**Figures 7B–D**). Furthermore, proteins involved in ferroptosis were analyzed by Western blot and qPCR. Compared with the COTI-2+EA group and COTI-2+Erastin+EA group, the COTI-2+Pantethine+EA group resulted in downregulation of TFR1 and ACSL4, while upregulating FTH1. Moreover, GPX4 and SLC7A11 were both markedly upregulated. Collectively, these changes contributed to the suppression of ferroptosis under the COTI-2+Pantethine+EA group (**Figures 7E, F**). These results further demonstrate that EA protects cardiomyocytes from rat under MIRI by inhibiting ferroptosis through the p53/SLC7A11/GPX4 pathway.

**Figure 7:**
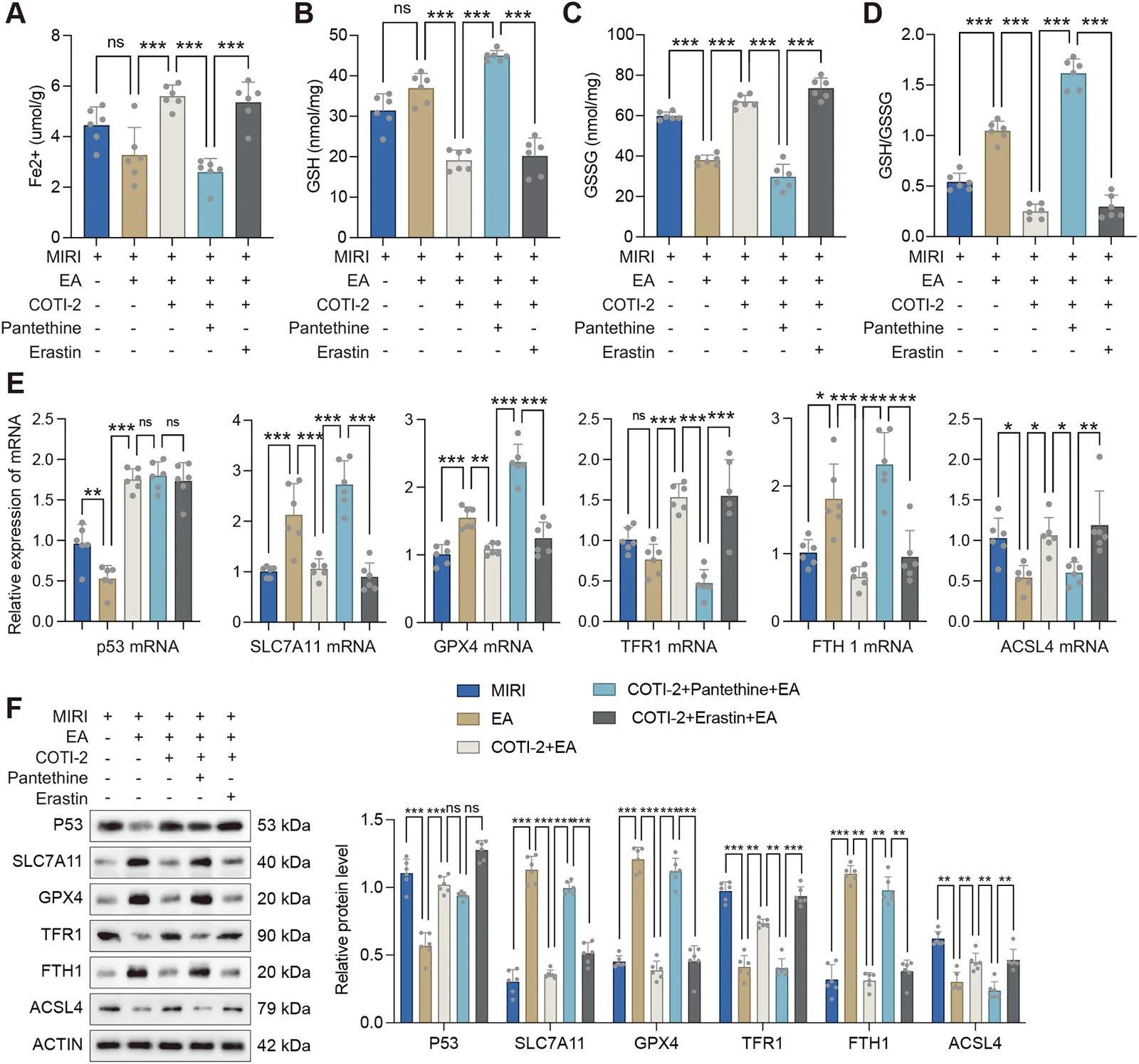
The SLC7A11 agonist reverses the pro-ferroptotic effects of the p53 agonist, confirming that electroacupuncture protects against MIRI via the p53/SLC7A11/GPX4 signaling pathway. **(A)** The Fe^2+^ content of cardiomyocytes in each group was quantified by the kit; **(B–D)** The GSH and GSSG contents of cardiomyocytes in each group were quantified; **(E)** The mRNA levels of proteins associated with ferroptosis in each group were identified again when the combination of p53 agonists and SLC7A11 agonists or SLC7A11 inhibitors were added. **(F)** The expression levels of proteins associated with ferroptosis in each group were once again identified. [Data are presented as Means ± SD of n = 6 independent biological replicates. Intergroup differences were assessed via one-way ANOVA followed by Tukey’s *post-hoc* multiple comparison test; *p < 0.05, **p < 0.01, ***p < 0.001, ns, not significant (*p*> 0.05)].

## 3 Discussion

A brief interruption of myocardial blood flow followed by timely reperfusion initiates a healing process in which the damaged myocardium is replaced by fibrotic scar tissue. This pathological remodeling further exacerbates cardiomyocyte apoptosis and fibrosis, ultimately contributing to progressive cardiac dysfunction (Prabhu and Frangogiannis, 2016). The severe injury resulting from reperfusion is termed MIRI. The development with MIRI is caused by the interaction of multiple mechanisms such as programmed cell death, lipid metabolism, glucose metabolism, oxidative stress, inflammation, and mitochondrial dysfunction, which could reach 50% of the total infarct area according to previous reports. Consequently, the development of a safer, multi-targeted, and more effective pretreatment strategy to mitigate MIRI is of urgent clinical importance.

Recent studies have demonstrated that among different forms of cellular demise, ferroptosis emerges as a pivotal contributor to MIRI development. Initially identified by Dixon in 2012, this distinct form of regulated cell death is characterized by excessive iron accumulation and ROS generation, leading to significant mitochondrial dysfunction. These pathological changes manifest as diminished or absent mitochondrial cristae, disruption of the outer mitochondrial membrane, increased membrane density, and compromised plasma membrane selectivity, collectively culminating in cellular demise (Yagoda et al., 2007; Yang and Stockwell, 2008; Cao and Dixon, 2016; Xie et al., 2016; Dixon, 2017; Latunde-Dada, 2017; Yu H. et al., 2017). Recent studies further highlight the pivotal role of mitochondrial morphological remodeling in MIRI-associated dysfunction. Research on type II diabetes with MIRI has revealed a significant reduction in mitochondrial fusion, while increased fission leads to markedly elevated levels of fragmented mitochondria. The accumulation of mitochondrial DNA within the cytoplasmic compartment leads to stimulation of the cGAS-STING signaling cascade, inducing TBK1 and IRF3 phosphorylation. This molecular event initiates a series of inflammatory reactions that aggravate MIRI (Xiong et al., 2023). Research focusing on acute renal injury has demonstrated that March6 protein exerts protective effects against ferroptosis in renal tubular epithelial cells through facilitating the breakdown of key ferroptosis-associated proteins, specifically ACSL4 and p53. This mechanism contributes to the amelioration of acute kidney injury progression (Feng et al., 2025). Meanwhile, during the process of diabetic with MIRI, Li et al. has demonstrated from epigenetic perspective that ALKBH5, a key enzyme in RNA demethylation, has upregulated G3BP1 expression through m6A modification. Subsequently, G3BP1 has interacted with YBX1 and p53, leading to reduced nuclear translocation of YBX1 and p53, resulting in a significant downregulation of p53-mediated transcriptional inhibition of SLC7A11, thereby inhibiting the ferroptosis process of cardiomyocytes and attenuating myocardial damage in diabetic with MIRI (Li et al., 2025).

Considering that suppressing the ferroptotic biochemical phenotype significantly alleviates ischemia-reperfusion damage in various organs such as the heart and kidneys, but effective clinical treatments for MIRI are still lacking, developing innovative therapeutic approaches has become imperative. For centuries, traditional Chinese medicine has been utilized in managing ischemic heart conditions, showing notable effectiveness in reducing myocardial ischemic symptoms and preventing disease recurrence. Consequently, the present research was designed to explore whether EA preconditioning could mitigate myocardial damage in MIRI rat models through suppressing the ferroptotic biochemical phenotype in cardiomyocytes (Hao et al., 2017; Feng et al., 2019).

Whether EA intervention could ameliorate oxidative damage in cardiomyocytes of rat with MIRI was first determined in this study. The results demonstrated that EA intervention alleviated energy metabolism disorders and oxidative damage in cardiomyocytes with MIRI. These findings are consistent with those reported by Yu et al, which indicated that melatonin administration enhanced the production of SOD and ATP, while reducing MDA generation, thereby attenuating myocardial injury (Yu L. et al., 2017). Intracellular iron homeostasis is maintained through a dynamic balance involving multiple pathways such as uptake, storage, release, and metabolism. Ferritin, composed of ferritin light chain (FTL) and FTH1 is involved in cellular iron storage. In this study, FTH1 expression was significantly downregulated in cardiomyocytes of MIRI rats, whereas EA intervention markedly restored FTH1 levels, suggesting that EA enhanced the iron storage capacity of cardiomyocytes and reduced intracellular Fe2+ accumulation. Moreover, ACSL4, a critical enzyme associated with phospholipid remodeling and a known driver of endogenous ferroptosis, was significantly decreased following EA intervention, consistent with previous findings on ferroptosis inhibition. GPX4 possesses crucial catalytic activity and plays an important role in inhibiting ferroptosis, and the GSH serves as an essential cofactor for GPX4. In this study, EA treatment significantly increased GSH levels in myocardial tissue, further supporting the conclusion that EA intervention mitigates the ferroptotic biochemical phenotype in cardiomyocytes and exerts a protective effect against MIRI-induced oxidative injury.

According to previous studies, the occurrence of cellular ferroptosis could be induced by multiple pathways, including exposure to high extracellular concentrations of glutamate, amino acid starvation, inhibition of the Xc-system, suppression of GPX4. Recently, accumulating evidence has revealed that the tumor suppressor protein p53, a DNA-binding transcription factor, also contributes to ferroptosis by transcriptionally regulating specific target genes. Among these, SLC7A11, which serves as the light chain subunit of the plasma membrane transporter System Xc^-^, mediates the uptake of cystine to maintain intracellular glutathione synthesis. Glutathione acts as the preferred substrate for the antioxidant enzyme GPX4, reducing cytotoxic lipid peroxides and thereby preserving cellular redox balance. Research has revealed that the p53 protein exhibits binding affinity for the SLC7A11 promoter region, effectively suppressing its transcriptional activity and consequently triggering ferroptosis in cells (Kang et al., 2019; Chen et al., 2021). Earlier studies have established that STAT6 plays a protective role against acute lung injury through ferroptosis inhibition, achieved by regulating the p53/SLC7A11 signaling pathway (Yang et al., 2022). In a related finding, MG53 administration was proven to mitigate cardiotoxic effects induced by doxorubicin, with its mechanism involving the p53/SLC7A11/GPX4 signaling axis to prevent ferroptosis (Jiang et al., 2024). Building upon these findings, our study further investigated whether EA suppresses ferroptosis in cardiomyocytes following MIRI through this signaling pathway.

In this study, it was found that after EA intervention, p53 protein was significantly down-regulated, SLC7A11 and GPX4 proteins were significantly upregulated, and enzymes related to cardiomyocyte injury were significantly downregulated in MIRI cardiomyocyte. Furthermore, when MIRI rat with EA intervention were treated with a p53 agonist or an SLC7A11 inhibitor, the anti-ferroptotic effect conferred by EA was abolished, and ferroptosis progression was exacerbated. These results indicated that EA modulates the ferroptosis process in MIRI cardiomyocytes via the p53/SLC7A11/GPX4 pathway, thereby exerting a protective effect on cardiomyocytes.

An outstanding mechanistic gap that merits in-depth discussion is how EA stimulation acts upstream to repress p53 expression and transcriptional function in injured myocardium. Based on existing preclinical acupuncture and cardiovascular research, two core regulatory axes may mediate EA-driven p53 suppression. First, EA stimulation at PC6, ST36 and CV4 with 2 Hz continuous waves activates somatic afferent nerve fibers, transmitting signals to the hypothalamic paraventricular nucleus (PVN) and nucleus tractus solitarius (NTS) to remodel the hypothalamic-pituitary-adrenal (HPA) and sympathetic-adrenal-medullary (SAM) neuroendocrine axes. As previously reported, EA targeting PVN CRH neurons can alleviate MIRI (Zhou et al., 2024); balanced neuroendocrine signaling attenuates ischemia-reperfusion-triggered p53 phosphorylation at Ser15 and Ser20, which prevents p53 protein stabilization and relieves its transcriptional repression of SLC7A11. Second, EA facilitates nuclear translocation of Nrf2, which forms reciprocal antagonistic crosstalk with p53 under oxidative stress (Yang et al., 2025). Activated nuclear Nrf2 not only directly upregulates SLC7A11 and GPX4 transcription to boost antioxidant capacity but also counteracts p53’s inhibitory binding to the SLC7A11 promoter, forming a coordinated dual-layer anti-ferroptotic regulatory network. Collectively, neuroendocrine reflex and Nrf2 redox signaling converge to mediate EA’s inhibitory effect on myocardial p53.

It should be noted that transmission electron microscopy(TEM) detection of shrunken mitochondria with decreased cristae and condensed membrane is the morphological gold standard for ferroptosis verification (Cao and Dixon, 2016; Yu H. et al., 2017; Xiong et al., 2023). Restricted by exhausted myocardial tissue and the tight revision schedule, we could not supplement TEM ultrastructural data in this work. Even so, our multi-dimensional biochemical results align closely with previously TEM-validated cardiac ferroptosis models (Dixon et al., 2012): the MIRI group displayed synchronized Fe²^+^ accumulation, GSH depletion, MDA elevation, upregulated pro-ferroptotic TFR1/ACSL4 and downregulated protective FTH1/GPX4. It is widely accepted in academia that significant simultaneous changes in the three biochemical indicators (Fe²^+^ accumulation, GSH depletion and elevated MDA) can form a reliable biochemical evidence chain confirming the existence of ferroptosis in the absence of TEM morphological data (Thi Nghiem et al., 2025). We further uncovered crosstalk between this iron-lipid peroxidation biochemical phenotype and mitochondrial apoptosis; lipid peroxidation products act as upstream stressors to trigger the Bax/Cleaved Caspase-3-mediated apoptotic cascade, and EA simultaneously suppresses both pathological signatures to confer cardioprotection.

Several inherent limitations of this study remain to be acknowledged. First, this work represents a proof-of-concept animal study with a relatively small biological sample size (n=6 per group). Although consistent statistical differences were detected across multiple biochemical and molecular readouts, conclusions drawn from the present dataset should be interpreted with caution, and larger-sample animal experiments are needed to consolidate these observations before definitive mechanistic inferences can be established. Second, the absence of TEM morphological evidence limits direct visualization of ferroptotic damage, and we will reserve adequate tissue for TEM observation in subsequent research to supply intuitive ultrastructural validation. Third, our cross-limb EA electrode wiring (same-side PC6–ST36) creates a transcardiac current loop, a key translational limitation inconsistent with human clinical safety standards requiring same-body-segment electrode pairing to avoid arrhythmia risks. Though this setup is widely adopted in rodent cardiac acupuncture research due to rodent anatomical constraints (Li et al., 2022), EA cardioprotection relies on somatic-autonomic neural reflexes instead of direct cardiac electrical interference. For clinical translation, identical acupoints (PC6, ST36, CV4) can be stimulated via clinic-compliant electrode schemes (e.g., pairing PC6 with a forelimb reference electrode) to retain equivalent neuroactivation without transcardiac current, which will be adopted in our future clinical trials.

In conclusion, Our proof-of-concept pre-clinical findings preliminarily demonstrate that EA pretreatment confers cardioprotection against MIRI, at least in part, by inhibiting the ferroptotic biochemical phenotype characterized by iron accumulation and lipid peroxidation via regulation of the p53/SLC7A11/GPX4 pathway. Further larger-sample animal and clinical investigations are warranted to validate these observations. Our animal findings offer preclinical molecular evidence to support the clinical application of EA preconditioning. The PC6/ST36/CV4 acupoint and low-frequency EA protocol adopted in this study aligns with the stimulation schemes widely used in human cardiac clinical trials. By targeting the p53/SLC7A11/GPX4 ferroptosis pathway, we uncovered a core molecular cascade explaining why EA effectively relieves perioperative myocardial injury observed in PCI and cardiac surgery patients. As an inexpensive, drug-free adjunct therapy without adverse reactions, EA preconditioning holds broad clinical prospects for high-risk cardiovascular patients. Further clinical research detecting ferroptosis-related blood biomarkers will help translate these preclinical results into standardized perioperative EA protective regimens.

## 4 Materials and methods

### 4.1 Animals and ethics

Animal and Ethical Standards: This study selected healthy, specific pathogen-free (SPF) male rats weighing 200–220 g, provided by Suzhou Xishan Biotechnology Co., Ltd. (license number: SYXK2023-0067). The experimental animals were kept in a barrier environment at the Experimental Animal Research Center of Hubei University of Chinese Medicine, with environmental conditions maintained at 20-24 °C, relative humidity of 45% -55%, and a 12 h light/dark cycle. The padding was changed every two days, and rats can freely obtain standard laboratory feed and drinking water. All rats were allowed to adapt to the environment 7 days before the experiment. This study was approved by the Animal Welfare and Ethics Committee of Hubei University of Chinese Medicine (approval number: HUCMS80674155), and all experimental procedures were performed in accordance with the National Institutes of Health’s “Guide for the Care and Use of Laboratory Animals” (1996 edition).

#### 4.1.1 Experimental design

Researchers randomly divided rats into nine groups using a random number table: blank control group (Ctrl), sham operation group (Sham), model group (MIRI), model combined with electroacupuncture pretreatment group (EA), model combined with RSL-3 (ferroptosis agonist) and electroacupuncture pretreatment group (RSL-3+EA), model combined with COTI-2 (p53 agonist) and electroacupuncture pretreatment group (COTI-2+EA), model combined with Erastin (SLC7A11 inhibitor) and electroacupuncture pretreatment group (Erastin+EA), model combined with COTI-2 and Erastin and electroacupuncture pretreatment group (COTI-2+Erastin+EA), model combined withCOTI-2 and pantethine (SLC7A11 agonist) and electroacupuncture pretreatment group (COTI-2+Pantethine+EA).

We adopted a classic pharmacological rescue experimental design to verify whether EA pretreatment alleviates MIRI-induced cardiomyocyte ferroptosis through the p53/SLC7A11/GPX4 signaling pathway. All agonist/inhibitor intervention groups in this study were set as EA + drug co-treatment rescue groups, which were used to reverse the cardioprotective phenotype of EA and verify the dependence of EA’s efficacy on the target pathway. Existing published studies have fully confirmed that separate administration of RSL-3 (ferroptosis agonist), COTI-2 (p53 agonist), or Erastin (SLC7A11 inhibitor) alone can aggravate myocardial ferroptosis and exacerbate myocardial ischemia/reperfusion injury, and their independent pro-ferroptotic and myocardial-damaging effects have been well documented in previous cardiovascular research (Park et al., 2019; Hou et al., 2025; Karampinos et al., 2026). Therefore, independent single-drug treatment groups without EA were not repeated in the present study to avoid redundant repetitive verification.

The experimental workflow is shown in **Supplementary Figure S1A**.

### 4.2 Construction of an animal model of MIRI

Sprague Dawley rats were anesthetized by intraperitoneal injection of pentobarbital sodium at a dose adjusted for body weight and then fixed on the operating table. After routine disinfection, insert them into endotracheal tubes and connect them to a ventilator to maintain breathing. Make a longitudinal incision of about 0.5 cm on the left side of the sternum, use hemostatic forceps to enlarge the intercostal space, and expose the left coronary artery and ventricle. Ligature the left anterior descending coronary artery and induce myocardial ischemia for 30 minutes. The normal electrocardiogram is shown in **Supplementary Figure S2A**, and the ST segment elevation during the ischemic period confirms the successful induction of myocardial infarction (**Supplementary Figure S2B**). Then release the ligature and allow the myocardium to reperfuse for 2 h, while maintaining continuous positive pressure ventilation until the end of the reperfusion phase. The electrocardiogram after 2 h of reperfusion is shown in **Supplementary Figure S2C**.

### 4.3 EA pre-treatment

Beginning seven days prior to MIRI induction, rats assigned to the EA pretreatment group received daily 20-minute EA stimulation. Take CV 4 (located on the midline of the abdomen, at the lower 1/5 point of the line connecting the upper edge of the pubic symphysis and the sternum xiphoid process, **Supplementary Figure S1B**),bilateral ST 36 (located on the lateral side of the lower leg of rats, about 5 cm below the depression of the posterolateral aspect side of the knee joint, between the tibialis anterior muscle and the peroneal longus muscle, **Supplementary Figure S1C**),and bilateral PC6 (located on the medial side of the forelimb of rats, about 3 mm above the median wrist crease, **Supplementary Figure S1D**), and use Suzhou produced No. 28 1-inch long needle (diameter 0.3mm, length 25mm) for electroacupuncture pretreatment. The needle was inserted to a depth of about 5 mm and connected to a HANS-200 electroacupuncture instrument. The same set of electrodes were connected to the PC6(negative pole) and ST36 (positive pole) on the same side, and CV4 acupoint (negative pole) was connected to add a needle (positive electrode) 5mm away from CV4 acupoint and connect it to the same set of electrodes. Use continuous wave with a frequency of 2 Hz and an intensity of 1 mA. The stimulation lasted for 20 min per session, once daily, for 7 consecutive days.

### 4.4 Pharmacological interventions

RSL3 (ferroptosis inducer): Dosage: 10 mg/kg; Solvent: 5% dimethyl sulfoxide (DMSO) diluted in sterile saline; Route of administration: Intraperitoneal injection; Timing & frequency: Once daily for 7 consecutive days, administered 60 min prior to daily EA stimulation;Dosage justification:The 10 mg/kg intraperitoneal dosage adopted herein is a standardized regimen widely applied in rat MIRI ferroptosis assays, which effectively blocks the antioxidant SLC7A11/GPX4 cascade (Dixon et al., 2012; Guo et al., 2024). COTI-2 (p53 activator):Dosage: 15 mg/kg; Solvent: 5% DMSO dissolved in sterile saline; Route of administration: Intraperitoneal injection; Timing & frequency: Once daily for 7 consecutive days; injected 60 min before daily EA stimulation;Dosage justification: The 15 mg/kg Intraperitoneal injection dose was selected based on previous publications demonstrating effective p53 activation in rodent models. Hou et al. (2025) reported that COTI-2 at 15 mg/kg Intraperitoneal injection significantly upregulated p53 expression and promoted ferroptosis in Cerebral Ischemia–Reperfusion Injury rat. This dose has been widely adopted in ferroptosis-related mechanistic studies and represents a well-established pharmacological intervention for p53 pathway activation *in vivo*.

Erastin (SLC7A11/Xc^-^ system inhibitor):Dosage: 20 mg/kg;Solvent: 5% DMSO dissolved in sterile saline;Route of administration: Intraperitoneal injection;Timing & frequency: Once daily for 7 consecutive days; injected 60 min before daily EA stimulation;Dosage justification: The 20 mg/kg intraperitoneal injection dose was based on established literature. Yang et al (Yang et al., 2020). and Hao et al (Tian et al., 2021). demonstrated that Erastin at 20 mg/kg intraperitoneal injection effectively inhibited System Xc^-^ and induced ferroptosis in rodent ischemia-reperfusion models. This dosage has been consistently employed as a standard SLC7A11 inhibition protocol in *in vivo* ferroptosis research.

Pantethine (SLC7A11 agonist):Dosage: 150mg/kg;Solvent: Dissolved directly in sterile saline (pantethine exhibits excellent aqueous solubility);Route of administration: Intraperitoneal injection;Timing & frequency: Once daily for 7 consecutive days; injected 60 min before daily EA stimulation;Dosage justification: The 150 mg/kg intraperitoneal dose of pantethine was adopted from Shi et al (Shi et al., 2025), who verified that this regimen effectively upregulated SLC7A11 and exerted anti-ferroptotic effects in glucocorticoid-induced osteoporosis murine models. Pantethine serves as a precursor of coenzyme A and enhances cystine uptake via SLC7A11 activation, thereby boosting GSH biosynthesis.

### 4.5 Detection of markers for cardiomyocytes oxidative and antioxidant levels

After the MIRI rat model was successfully prepared, cardiac tissues were rapidly isolated and stored frozen at -20 °C. Fresh cardiac tissue samples weighing 0.1 g were homogenized using phosphate-buffered saline (PBS) and cell lysis buffer. Following centrifugation, the resulting supernatant was collected for subsequent analysis of various oxidative stress and antioxidant indicators using specific commercial assay kits. For ATP quantification, the Beyotime ATP Assay Kit was employed following the manufacturer’s instructions. Standard calibration samples were prepared, and the ATP detection working solution was freshly prepared prior to analysis. This working solution was dispensed into both experimental and control wells. The enzymatic reaction between ATP in the samples, firefly luciferase, and luciferin produced measurable luminescence, which was detected and quantified using a chemiluminescence measurement system. Myocardial malondialdehyde (MDA) content was assessed using Beyotime’s Lipid Peroxidation (MDA) Assay Kit. This method relies on the formation of a red-colored MDA-thiobarbituric acid (TBA) adduct under high-temperature acidic conditions, with absorbance measurements performed at 532 nm using a microplate reader. Superoxide dismutase (SOD) enzymatic activity was evaluated with Beyotime’s SOD Activity Assay Kit, which utilizes the water-soluble tetrazolium salt (WST-8) methodology for detection. Superoxide anions generated by the xanthine oxidase system react with WST-8 to form a formazan dye. Because SOD catalyzes the dismutation of superoxide anions, the production of formazan is inhibited proportionally to SOD activity. SOD activity was quantified by colorimetric detection of the WST-8 reaction product. Total glutathione content was measured at 412 nm based on the reduction of oxidized glutathione (GSSG) to reduced GSH by glutathione reductase, followed by the reaction of GSH with DTNB to form yellow-colored TNB and regenerate GSSG. After quantifying GSSG, the GSH level was calculated by subtracting GSSG from the total glutathione content.

### 4.6 Measurement of ROS levels using DCFH-DA probe

Fresh cardiac tissue (0.1 g) was collected and homogenized in PBS and cell lysis buffer.

Following centrifugation, the liquid supernatant was removed, and the remaining pellet was reconstituted in PBS to prepare a cell suspension. The DCFH-DA fluorescent probe underwent dilution with PBS at a 1:1000 concentration ratio before being introduced into the cell suspension mixture. This mixture was then maintained at 37 °C for a duration of 20–30 minutes under light-protected conditions to facilitate complete cellular uptake of the probe. Within the intracellular environment, esterase enzymes catalyzed the conversion of DCFH-DA to DCFH, which then underwent oxidation through interaction with reactive oxygen species (ROS), yielding the fluorescent product DCF. The measured fluorescence intensity demonstrates a direct correlation with the concentration of intracellular ROS. An appropriate amount of PBS resuspend the cells, adjust a certain concentration of cell suspension, and measure the ROS content by flow cytometry.

### 4.7 Determination of Fe2+ levels in cardiomyocytes

A total of 0.1 g of freshly excised cardiac tissue was processed by homogenization using PBS containing cell lysis buffer. After centrifugation, the resulting supernatant was removed, and the remaining pellet was reconstituted in PBS to prepare a cellular suspension. This suspension was subsequently transferred into a 96-well microplate. The samples were treated with RhoNox-6 reagent and maintained at 37 °C under light-protected conditions for a duration of 30 minutes. Fe²^+^ in the samples specifically binds to RhoNox-6, resulting in red fluorescence that is detected using a fluorescence microplate reader.

### 4.8 Western blot

Each group of heart tissue was collected into EP tubes, PBS and cell lysate were added for lysis on ice with sonication. The supernatant was absorbed and transferred to a new EP tube via centrifugation at 4 °C, which was added 1/4 volume of protein loading buffer according to the volume of the supernatant and then boiled for 10 min to completely denature the protein. The prepared protein samples were stored at -20 °C. An electrophoretic gel (consisting of a separating gel and a stacking gel) was prepared. Each group of samples were added to each gel lane hole, and the protein sample was electrophoresed to the lowest layer of the separation gel to be transferred. The PVDF membrane was cut into an appropriate size for the transfer process. The electrophoretic gel, PVDF membrane and filter paper were placed in the membrane conversion device, and the membrane conversion tank was placed in the ice-water mixture to maintain the constant pressure of 100 v membrane conversion for 90 min at a low temperature. After the transfer was completed, the PVDF membrane was incubated with specific antibodies, and finally the chemiluminescent substrate was added and the PVDF membrane was placed on a chemiluminescence imager for collecting experimental data.

### 4.9 RT-PCR analysis

Cardiac tissue samples were placed in EP tubes and processed by homogenization followed by centrifugation using 1 mL of Trizol reagent. The resulting supernatant was carefully pipetted into fresh centrifuge tubes, with each tube receiving 200 μL of chloroform solution. Following thorough vortexing, the samples underwent another centrifugation step. The RNA-containing aqueous phase was then transferred to new tubes containing isopropanol for precipitation. After subsequent centrifugation, the supernatant was removed, and the RNA pellet was resuspended in 60 μL of DEPC-treated water to ensure complete dissolution. The extracted RNA was preserved at -80 °C for long-term storage. Following the manufacturer’s protocol, appropriate quantities of RNA were subjected to reverse transcription using the specified reaction conditions, thereby converting the RNA templates into cDNA.

The primer sequence was shown in **Table 1** below.

**Table 1:** Real-time PCR primer sequences.

Name	Forward primer (5’ to 3’)	Reverse primer (5’ to 3’)
FTH1	CCCCCATTTGTGTGACTTCAT	GCCCGAGGCTTAGCTTTCATT
TFR1	ACCATTGTCATATACCCGGTTCA	CAATAGCCCAAGTAGCCAATCAT
ACSL4	CATCCCTGGAGCAGATACTCT	TCACTTAGGATTTCCCTGGTCC
BAX	CCCGAGAGGTCTTTTTCCGAG	CCAGCCCATGATGGTTCTGAT
BCL2	GGTGGGGTCATGTGTGTGG	CGGTTCAGGTACTCAGTCATCC
Caspase 3	CATGGAAGCGAATCAATGGACT	CTGTACCAGACCGAGATGTCA
P53	CAGCACATGACGGAGGTTGT	TCATCCAAATACTCCACACGC
SLC7A11	TCTCCAAAGGAGGTTACCTGC	AGACTCCCCTCAGTAAAGTGAC
GPX4	GAGGCAAGACCGAAGTAAACTAC	CCGAACTGGTTACACGGGAA

### 4.10 Determination of myocardial injury markers

After reperfusion of myocardial ischemic rat models in each group, the femoral vein was punctured to collect blood to detect myocardial injury markers. Serum was separated by centrifugation at 5000 × g for 20 min at 4 °C. The levels of cTnI, CK, and LDH were identified through the rat cardiac troponin I (cTnI) enzyme-linked immunosorbent assay (ELISA) kit, creatine kinase (CK) activity detection kit and lactate dehydrogenase (LDH) activity detection kit based on steps described in the manufacturer’s instructions.

### 4.11 Detection of cardiomyocyte apoptosis via TUNEL staining

Fresh heart tissue in each group was collected into EP tubes, PBS and cell lysate were added for lysis. Following centrifugation at 1000 g, the supernatant was carefully removed. The specimens underwent fixation using 4% paraformaldehyde for a duration ranging from 30 to 60 minutes. Subsequently, they were treated with PBS solution containing 0.1% Triton X-100 for 2 minutes under ice-cold conditions, followed by another PBS washing step. The prepared TUNEL reagent mixture (comprising TdT enzyme and FITC-dUTP) was applied to the samples, which were then maintained at 37 °C for one hour. Upon completion of this incubation period, excess fluorescent dye was eliminated through PBS washing. Finally, propidium iodide (PI) staining solution was introduced, and the samples were kept at ambient temperature for 10–20 minutes while being shielded from light exposure. Appropriate channels were set up to detect the fluorescence signals of FITC and PI, and flow cytometry was used to analyze the apoptosis ratio.

### 4.12 Statistical analysis

Statistical analyses were performed with GraphPad Prism 8 (GraphPad Software, USA). One-way ANOVA followed by Tukey’s post-hoc test was used for multi-group comparisons. Data are presented as Means ± SD, n = 6 independent biological replicates per group. *p < 0.05, **p < 0.01, ***p < 0.001; ns, not significant.

## Data Availability

The original contributions presented in the study are included in the article/**Supplementary Material**, further inquiries can be directed to the corresponding authors.
