# Supplementary material for: Electroacupuncture pretreatment attenuates myocardial ischemia/reperfusion injury-induced ferroptosis via the p53/SLC7A11/GPX4 pathway
Source: Front Physiol. 2026 Oct 12;17:1928208. doi: 10.3389/fphys.2026.1928208 (PMC13696320; doi:10.3389/fphys.2026.1928208)
Supplement: Supplementary file 1 [file Supplementaryfile1.docx]

**Supply materials**

**Figure S1** Experiments protocols and acupoint locations. A Experiments protocols.B Location of CV4 (Guanyuan) acupoint. C Location of ST36 (Zusanli) acupoint. DLocation of PC6 (Neiguan) acupoint

**Figure S2** Changes in electrocardiogram of rats during the modeling process.A is a normal electrocardiogram, B is an electrocardiogram after 30 minutes of ischemia, and C is an electrocardiogram after 120 minutes of reperfusion.
